# Comparative Analysis of the Complete Chloroplast Genomes of Eight *Salvia* Medicinal Species: Insights into the Deep Phylogeny of *Salvia* in East Asia

**DOI:** 10.3390/cimb47070493

**Published:** 2025-06-27

**Authors:** Yan Du, Yang Luo, Yuanyuan Wang, Jiaxin Li, Chunlei Xiang, Meiqing Yang

**Affiliations:** 1 Baotou Medical College, Baotou 014040, China; 102014126@btmc.edu.cn (Y.D.); 13573717323@163.com (Y.W.); xinxins163@163.com (J.L.); 2 Kunming Institute of Botany, Chinese Academy of Sciences, Kunming 650201, China; luoyang@mail.kib.ac.cn (Y.L.); xiangchunlei@mail.kib.ac.cn (C.X.)

**Keywords:** *Salvia*, chloroplast genome, comparative analysis, phylogenetic relationships

## Abstract

*Salvia*, a medicinally and economically important genus, is widely used in traditional medicine, agriculture, and horticulture. This study compares the chloroplast genomes of eight East Asian *Salvia* species to assess genetic diversity, structural features, and evolutionary relationships. Complete chloroplast genomes were sequenced, annotated, and analyzed for gene content, codon usage, and repetitive sequences. Phylogenetic relationships were reconstructed using Maximum Likelihood, Maximum Parsimony and Bayesian inference. The genomes exhibited a conserved quadripartite structure (151,081–152,678 bp, GC content 37.9–38.1%), containing 114 unique genes with consistent arrangement. Codon usage favored A/T endings, with leucine (Leu) most frequent and cysteine (Cys) least. We identified 281 long sequence repeats (LSRs) and 345 simple sequence repeats (SSRs), mostly in non-coding regions. Comparative analysis revealed five hypervariable regions (*trnH*-*psbA*, *rbcL*-*accD*, *petA*-*psbJ*, *rpl32*-*trnL*, *ycf1*) as potential molecular markers. Phylogenetic analysis confirmed the monophyly of East Asian *Salvia*, dividing them into five clades, with Sect. *Sonchifoliae* basal. While G1, G3, and G8 were monophyletic, G5 and G6 were paraphyletic, and the G7-G8 relationship challenged traditional classifications. The genomic evidence provides crucial insights for resolving long-standing taxonomic uncertainties and refining the classification system of *Salvia*. These findings suggest a complex evolutionary history involving hybridization and incomplete lineage sorting, providing valuable genomic insights for *Salvia* phylogeny, taxonomy, and conservation.

## 1. Introduction

Chloroplasts are vital organelles responsible for photosynthesis in plants, converting solar energy into carbohydrates essential for plant growth and development [1]. The chloroplast (cp) genome typically comprises a single circular DNA molecule with a highly conserved quadripartite structure, including a large single-copy (LSC) region, a small single-copy (SSC), and two inverted repeat (IR) regions, which contribute to genome size variation. The chloroplast genome of higher plants is generally between 120 and 160 kb in length, which is highly conserved in gene structure and order, encoding approximately 110–130 genes. According to the function of genes, we can roughly classify them into two categories: central cellular process genes including those involved in photosynthesis, transcription, and translation, along with specialized functional genes such as *matK* (RNA processing), *cemA* (membrane transport), *accD* (lipid biosynthesis), and *ccsA* (cytochrome assembly) [2,3]. The chloroplast genome is generally more tractable for comparative analyses than the mitochondrial genome due to its structural stability (conserved quadripartite architecture), higher sequence conservation, and absence of RNA editing, in addition to advantages over nuclear genomes such as moderate size, maternal inheritance, and strong collinearity across species [4]. With the advent of high-throughput sequencing technologies, research on chloroplast genome evolution has expanded rapidly [5], establishing chloroplast genomes as valuable resources for phylogenetic and evolutionary studies.

The genus *Salvia* L. (Lamiaceae), the largest genus in the mint family, comprises nearly 1000 species of annual or perennial herbs or small shrubs distributed across tropical and temperate regions of the world [6]. The genus holds significant cultural, medicinal, and economic importance. In China, *Salvia* species have been traditionally used to treat hepatic, renal, cardiovascular, and immune-related diseases [7]. In Europe and America, they serve primarily as flavoring agents, while in Africa, some species are used to treat conditions such as malaria and cancer [8]. Additionally, *Salvia* species are ornamental plants with fragrance, used not only for the processing of spices but also cultivated as ornamental plants and used in cosmetics, perfumery, landscaping, and aromatherapy [9]. China is the diversity center of *Salvia* in East Asia, harboring approximately 82 species out of the ~100 species in the region [10]. However, the taxonomic classification of *Salvia* species remains challenging due to extensive morphological variation and overlapping traits. Traditionally, *Salvia* was divided into three subgenera based on floral and stamen morphology: subg. *Salvia* (bilabiate flowers and glandular structures), subg. *Sclarea* (unique stamen structure, floral morphology, and herbaceous habit) and subg. *Allagospadonopsis* (distinct stamen morphology) [11]. Recent molecular phylogenetic studies using chloroplast gene fragments (e.g., *rbcL*, *matK*, *ycf1*) and nuclear markers (e.g., *ITS*), have led to a reassessment of the classification of *Salvia* in China [12]. The subsequent studies (*psbA–trnH*, *ycf1–rps15*, *trnL–trnF*, *rbcL*, *ITS* and *ETS*) have revealed complex evolutionary patterns and led to the proposal of new subgenera such as subgenus *Glutinaria*, which comprises all East Asian species [13], along with others like *Calosphace* in the Americas [14]. East Asian *Salvia*, once assigned to subg. *Salvia*, is now recognized as a distinct monophyletic group containing eight subclades (G1–G8) [13]. Despite this reclassification, phylogenetic relationships among these subclades remain ambiguous, often due to limited sampling and insufficient resolution from conventional DNA markers [15].

Complete chloroplast genome sequencing offers higher resolution for reconstructing phylogenies and identifying molecular markers than single or multi-locus datasets [16]. In this study, we sequenced and assembled the chloroplast genomes of eight East Asian *Salvia* species (*Salvia sonchifolia* C. Y. Wu, *Salvia wardii* Stib., *Salvia roborowskii* Maxim., *Salvia przewalskii* Maxim., *Salvia plebeia R. Br.*, *Salvia kiangsiensis* C. Y. Wu, *Salvia trijuga* Diels, and *Salvia chienii* Stib through the Illumina sequencing platform. Our specific aims were to (1) characterize their chloroplast genome structures and genetic features, (2) identify potential molecular markers for species delimitation and phylogenetic studies, and (3) reconstruct a high-resolution phylogeny of East Asian *Salvia*. These findings enhance our understanding of chloroplast genome evolution and provide critical data for taxonomic, evolutionary, and conservation research in this medicinally and ecologically significant genus.

## 2. Materials and Methods

### 2.1. Plant Material, DNA Extraction, and Sequencing

Fresh leaves of eight *Salvia* species (*S. sonchifolia*, *S. wardii*, *S. roborowskii*, *S. przewalskii*, *S. plebeia*, *S. kiangsiensis*, *S. trijuga*, and *S. chienii*) were collected from different localities (Table 1) and immediately dried using silica gel to preserve DNA integrity. Voucher specimens were deposited at the herbarium of the Kunming Institute of Botany (KUN), Chinese Academy of Sciences. Total genomic DNA was extracted using the modified cetyltrimethylammonium bromide (CTAB) method described by Doyle (1987) [17] and quantified using a NanoDrop spectrophotometer (Thermo Fisher Scientific, Wilmington, DE, USA) to ensure appropriate quality and concentration. Sequencing libraries were constructed and sequenced on the Illumina HiSeq X Ten platform, producing 150 bp paired-end reads. Sequencing was performed by Novogene (Beijing, China). The resulting chloroplast genome sequences were deposited in GenBank under the accession numbers listed in Table 1. The species highlighted in bold constitute the primary focus of this study.

### 2.2. Chloroplast Genome Assembly and Annotation

Raw sequencing reads were first processed using Fastp (v 0.23.1) to perform quality control, including adapter removal, trimming of low-quality bases (<Q15), and discarding short reads (<15 bp). High-quality sequencing reads were assembled into complete chloroplast genomes using the GetOrganelle pipeline [18], which is optimized for accurate de novo assembly of organellar genomes. Assemblies were generated using default parameters, with the *Salvia miltiorrhiza* chloroplast genome (GenBank accession: JX312195) serving as a reference. Circularity and assembly completeness were validated via BLAST (https://blast.ncbi.nlm.nih.gov/, accessed on 1 May 2022), confirming structural integrity and high similarity to the reference genome.

Manual curation and visualization were conducted using Geneious v10.2.2 (Biomatters Ltd., Auckland, New Zealand), ensuring accurate genome annotation and boundary definitions. Genome annotation was performed using the Plastid Genome Annotator (PGA; https://github.com/quxiaojian/PGA, accessed on 15 May 2022) [19], with start/stop codons and intron-exon boundaries manually verified in Geneious v10.2.2. tRNA genes were further confirmed using tRNAscan-SE v2.0 [20]. Circular genome maps were generated using OGDRAW v1.2 [21] (https://chlorobox.mpimp-golm.mpg.de/OGDraw.html, accessed on 1 June 2022), displaying gene arrangement and GC content.

### 2.3. Genome Structure and Codon Usage Analysis

Chloroplast genome structures, including the large single-copy (LSC), small single-copy (SSC), and inverted repeat (IR) regions, were analyzed using Repeat Finder in Geneious v10.2.2. Basic genomic features such as genome length, GC content, and region sizes were compared across species. To evaluate codon usage bias, Relative synonymous codon usage (RSCU) values for protein-coding regions were calculated using MEGA X v10.0.5. RSCU measures the normalized usage frequency of a specific codon relative to other synonymous codons encoding the same amino acid. An RSCU value > 1 indicates that the codon is used more frequently than expected, suggesting a strong preferential usage in the species. Conversely, an RSCU value < 1 implies that the codon is used less frequently, reflecting weaker preference or avoidance. When RSCU = 1, the codon is used at the expected frequency with no apparent bias.

### 2.4. Long and Simple Sequence Repeats Analyses

Long sequence repeats (LSRs), including palindromic, reverse, forward, and tandem repeats, were identified using REPuter [22] with a minimum repeat size of 30 bp and a sequence identity threshold of ≥90% (i.e., Hamming distance was 3). Meanwhile, tandem repeats were further validated using Tandem Repeats Finder v4.07b [23]. The analysis was performed using default parameters with alignment scores weighted at match = 2, mismatch = 7, and indels = 7, a minimum alignment score threshold of 50 for reporting repeats, and a maximum period size of 500 bp.

Simple Sequence Repeats (SSR) were predicted with MISA v2.1 (http://pgrc.ipk-gatersleben.de/misa/misa.html, accessed on 8 October 2022) [24] under the following thresholds: mononucleotide repeats ≥ 10, dinucleotide repeats ≥ 5, trinucleotide repeats ≥ 4, tetranucleotide repeats ≥ 3, pentanucleotide repeats ≥ 3, and hexanucleotide repeats ≥ 3. Finally, all repeat sequence prediction results were manually corrected to remove redundant parts. Specifically, the raw output from MISA was subjected to manual curation to eliminate redundancy and false positives. We visually inspected the results in a genome browser to verify the repeat structures. Redundant results were handled by merging compound SSRs (i.e., two or more SSRs adjacent to each other with a spacing of <100 bp) into a single complex SSR locus for statistical purposes. For example, an (AT)6 motif immediately followed by a (GT) 5 motif was treated as one compound SSR, not two separate SSRs. This manual verification and correction step ensured an accurate and non-redundant dataset for all subsequent comparative analyses.

### 2.5. Comparative Genomic Analysis

Comparative analysis of the eight *Salvia* chloroplast genomes was conducted using the mVISTA tool with the Shuffle-LAGAN alignment mode [25], using *S. roborowskii* as the reference. Alignments were visualized to assess sequence conservation and divergence. The boundaries of LSC, SSC, and IR regions were compared to detect structural variation due to IR expansion and contraction. Focus was placed on genes often affected by IR shifts, including *rps19*, *ndhF*, and *ycf1* [26].

Sliding window analysis was performed using DnaSP v5 [27] to compute nucleotide diversity (Pi) values across the chloroplast genomes. The analysis employed a window size of 600 bp and a step size of 200 bp, enabling the identification of highly variable regions. These regions were evaluated for their potential as molecular markers for phylogenetic studies.

### 2.6. Phylogenetic Analyses

Complete chloroplast genome sequences of 39 *Salvia* species, including the eight newly sequenced species, were used for phylogenetic reconstruction. Additional sequences were retrieved from GenBank, as listed in Table 1. The sequences were aligned using MAFFT v1.5.0 with the alignment strategy set to “Auto”, followed by visual inspection of the alignments in Geneious Prime. To ensure data quality, only terminal sequencing artifacts—approximately 50 base pairs from both the 5′ and 3′ ends of each sequencewere removed. Phylogenetic analyses were subsequently conducted on the whole cp genome. Phylogenetic trees were constructed using maximum parsimony (MP) in PAUP* v.4.0b10 [28], maximum likelihood (ML) in RAxML-HPC BlackBox on the CIPRES Science Gateway [29], and Bayesian inference (BI) in MrBayes v3.2.7 [30]. For MP analysis, all characters were treated as unordered and equally weighted, with gaps coded as missing. A heuristic search was implemented with tree-bisection-reconnection (TBR) branch swapping and 1000 random addition sequence replicates. Clade support values were assessed by performing 1000 bootstrap replicates. The nucleotide substitution models for both ML and BI analyses were selected through rigorous statistical evaluation using ModelFinder as implemented in IQ-TREE 2 (v2.2.0). The best-fitting model of molecular evolution (GTR + GAMMA) was determined using the Akaike Information Criterion (AIC). For ML analysis, we set a bootstrap of 1000 replicates. For BI analysis, the MCMC analysis included four chains (one cold and three heated) running for 1,000,000 generations, with trees sampled every 1000 generations. Convergence was monitored through the average standard deviation of split frequencies (<0.01). The first 25% of sampled trees were discarded as burn-in, and the remaining trees were used to construct a majority-rule consensus tree.

## 3. Results

### 3.1. Size and Structure of Chloroplast Genomes

**Figure 1:**
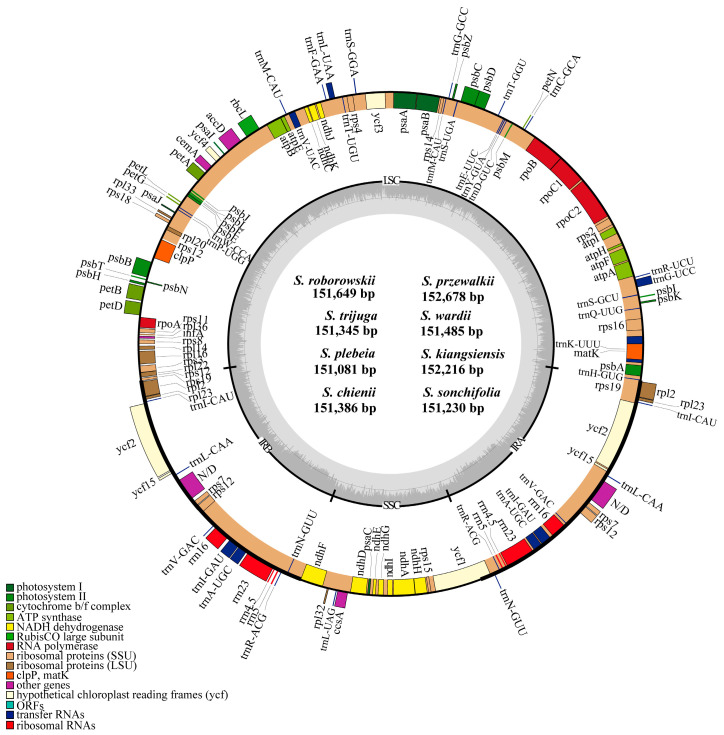
Chloroplast genome map of eight *Salvia* species. The circular map depicts a representative plastome (*Salvia roborowskii*). Thick lines on the outer complete circle represent the inverted repeat regions (IRa and IRb). The innermost track of the plastome shows the GC content. Genes on the outside of the map are transcribed in a clockwise direction, and genes on the inside of the map are transcribed in a counterclockwise direction. IR, inverted repeats; LSC, large single-copy; SSC, small single-copy.

The chloroplast genomes of the eight *Salvia* species exhibited a conserved quadripartite structure, comprising a large single-copy (LSC) region (82,464–83,912 bp), a small single-copy (SSC) region (17,493–17,638 bp), and two inverted repeat (IR) regions (25,321–25,596 bp each). Genome sizes ranged from 151,081 bp (*S. plebeia*) to 152,678 bp *(S. przewalskii*), with overall GC content ranging from 37.9% to 38.1% (Figure 1). Among the three regions, the IR regions exhibited the highest GC content (43.1–43.2%) due to the presence of rRNA genes, while the LSC (36.0–36.3%) and SSC (31.7–32.0%) regions had lower GC content. The chloroplast genomes of all eight *Salvia* species contained 132 unique genes including 80 protein-coding regions (CDS), 30 tRNA genes, four rRNA genes, and 18 genes duplicated within the IR regions. These duplicated genes included seven protein-coding genes (*rpl2*, *rpl23*, *ycf2*, *ycf15*, *ndhB*, *rps7,* and *rps12*), seven tRNA genes (*trnI-CAU*, *trnL-CAA*, *trnV-GAC*, *trnI-GAU*, *trnA-UGC*, *trnR-ACG,* and *trnN-GUU*), and all four rRNA genes (*rrna16*, *rrna23*, *rrna4.5,* and *rrna5*). There were 18 intron-containing genes, of which 15 genes (*atpF*, *rpoC1*, *ndhB*, *petB*, *petD*, *rpl2*, *rpl16*, *rps16*, *ndhA*, *trnA-UGC*, *trnI-GAU*, *trnK-UUU*, *trnL-UAA*, *trnG-UCC,* and *trnV-UAC*) had a single intron, while the other three (*clpP*, *ycf3,* and *rps12*) contained two introns (Table 2 and Table 3).

### 3.2. Codon Usage Preference Analysis

**Figure 2:**
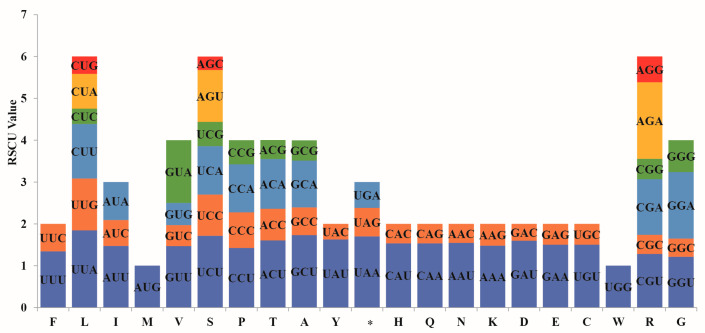
Relative synonymous codon usage (RSCU) values in the genus *Salvia*, taking *S. sonchifolia* as an example. F: phenylalanine; L: leucine; I: isoleucine; M: methionine; V: valine; S: serine; P: proline; T: threonine; A: alanine; Y: tyrosine; *: stop; H: histidine; Q: glutamine; N: asparagine; K: lysine; D: aspartic acid; E: glutamic acid; C: cysteine; W: tryptophan; R: arginine; G: glycine.

The codon usage patterns among the eight *Salvia* species were highly conserved, exhibiting only minor variations in absolute codon counts, see Table S1. Across all chloroplast genomes, a total of 64 codons were identified, of which 61 encoded amino acids and three represented stop codons. Among these, *UGG* (Trp) and *AUG* (Met) exhibited no usage bias, while the remaining 62 codons displayed consistent preferences across species. Thirty codons were used more frequently (RSCU > 1), including *UUU*, *UUG*, *CUU*, *AUU*, *GUU*, *GUA*, *UCU*, *UCA*, *CCU*, *CCA*, *ACU*, *ACA*, *GCU*, *GCA*, *UAU*, *UAA*, *CAU*, *CAA*, *AAU*, *AAA*, *GAU*, *GAA*, *UGU*, *CGU*, *CGA*, *AGU*, *GGU*, *GGA*, *UUA,* and *AGA*. Notably, UUA (Leu) and AGA (Arg) were among the most preferred codons. In contrast, 32 codons were used less frequently (RSCU < 1), including *UUC*, *CUC*, *CUA*, *CUG*, *AUC*, *AUA*, *GUC*, *GUG*, *UCC*, *UCG*, *CCC*, *CCG*, *ACC*, *ACG*, *GCC*, *GCG*, *UAC*, *UAG*, *CAC*, *CAG*, *AAC*, *AAG*, *GAC*, *GAG*, *UGC*, *UGA*, *CGC*, *CGG*, *AGC*, *AGG*, *GGC*, *GGG.* Among preferred codons, 13 codons ended with adenine (A), 16 with uracil (U), whereas only one ended with guanine (G), and none with cytosine (C) (Figure 2). This strong preference for A/U at the third codon position (96.7% of preferred codons) reflects the overall AT-rich composition of *Salvia* chloroplast genomes and is consistent with codon usage patterns observed in other angiosperms [31].

Taking *Salvia sonchifolia* as a representative example, the total number of codons in protein-coding genes was 22,716. The most frequently used codon was AUU (Ile, 956 occurrences; RSCU = 1.49), whereas UGC (Cys, 55 occurrences; RSCU = 0.45) was the least used. Among amino acids, leucine showed the highest occurrence (2401 codons; 10.6%), with a strong preference for AT-ending codons such as UUA (31.9%) and CUU (21.7%). In contrast, cysteine was the least represented amino acid (1.08%). This conserved codon usage pattern was consistent across all seven other species, with leucine maintaining remarkably consistent representation (10.3–10.6% in *S. roborowalskii*, *S. przewalskii*, *S. trijuga*, *S. wardii*, *S. plebeia*, *S. kiangsiensis,* and *S. chienii*) and cysteine remaining the least (1.1%).

### 3.3. Long and Simple Sequence Repeats Analyses

**Figure 3:**
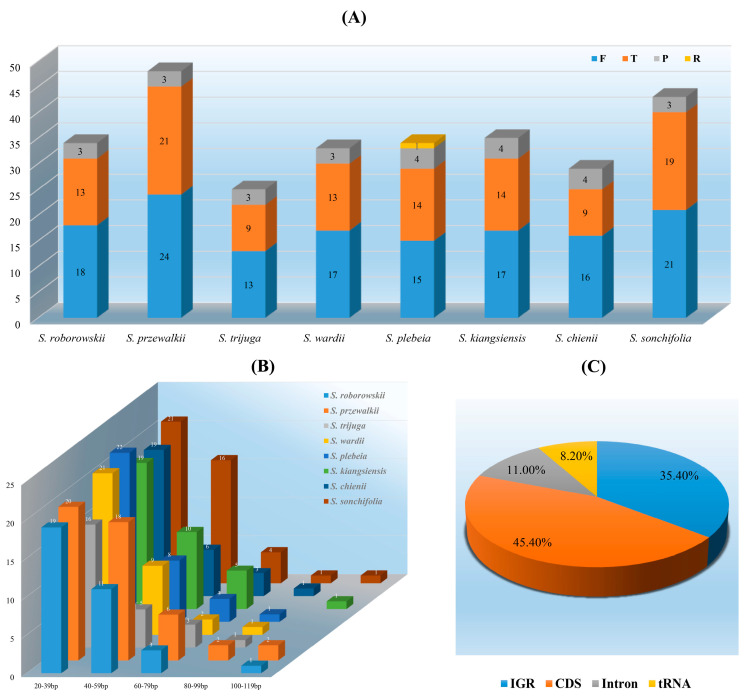
Types, lengths, and distributions of long sequence repeats in the eight *Salvia* chloroplast genomes. (**A**) Number of different repeat types: F, forward; P, palindromic; R, reverse; T, tandem; (**B**) Number of different repeat lengths; (**C**) Proportion of repeats in LSC, SSC, and IR regions.

Long sequence repeats (LSRs) play a critical role in chloroplast genome evolution contributing to structural rearrangements, genome expansion, and recombination events. [32]. Across the eight *Salvia* species examined, a total of 281 LSRs were identified including 141 forward repeats, 27 palindromic repeats, 112 tandem repeats, and one reverse repeat. *S. przewalskii* contained the highest number of repeats (48), while *S. trijuga* had the fewest (25) (Figure 3A). The majority of LSRs (85.4%) were shorter than 60 bp, while repeats longer than 100 bp accounted for only 1.8% (Figure 3B). Regionally, LSRs were predominantly located in protein-coding regions (CDS; 45.4%), followed by intergenic regions (IGR; 35.4%) and intron regions (8.2%) (Figure 3C). Shared repeat sequences were identified in various genomic regions, including IGR (*rps12-trnV-GAC* and *rrn4.5-rrn5*), tRNA genes (*trnG-GCC*, *trnG-GGA*, *trnS-GC,* and *trnS-GGA*), the *ndhA* intron, and coding regions (*ycf1* and *ycf2*) (Table 4). Most of these repeats were forward or tandem types and are likely involved in maintaining chloroplast genome structure and facilitating evolutionary conservation.

**Figure 4:**
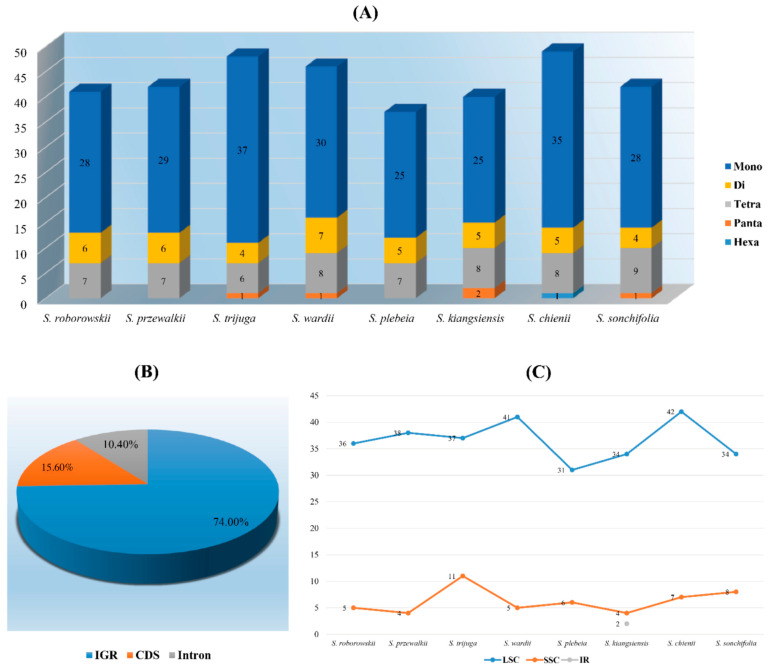
The number and distribution of SSRs in the chloroplast genomes of eight *Salvia* species. (**A**) Total number of repeats; (**B**) Proportion of repeats in IGR, CDS, or Intron regions; (**C**) Number of repeats in LSC, SSC, and IR.

In addition to LSRs, simple sequence repeats (SSRs), also known as microsatellites, were analyzed for their potential utility as genetic markers. Across the eight *Salvia* species examined, a total of 345 SSRs were identified, with the number ranging from 37 in *S. plebeia* to 49 in *S. chienii*. Mononucleotide repeats dominated the SSR profiles (>68.7%), with counts ranging from 25 to 37 (Figure 4A). The majority of mononucleotide repeats were polyadenine (polyA) and polythymine (polyT), making up 42.2% and 56.1%, respectively, while ployguanine (polyG) or polycytosine (polyC) repeats were relatively rare. Hexanucleotide repeats were only detected in *S. chienii*, and no trinucleotide repeats were observed in any species. The majority of SSRs were located in intergenic regions (IGR, 74.0%), followed by protein-coding regions (CDS, 15.6%) and intron regions (10.4%) (Figure 4B). Regional distribution analysis revealed that the LSC region harbored the highest proportion of SSRs (84.9%), whereas the SSC and IR regions contained relatively fewer repeats (Figure 4C).

### 3.4. Inverted Repeat Expansion and Contraction

**Figure 5:**
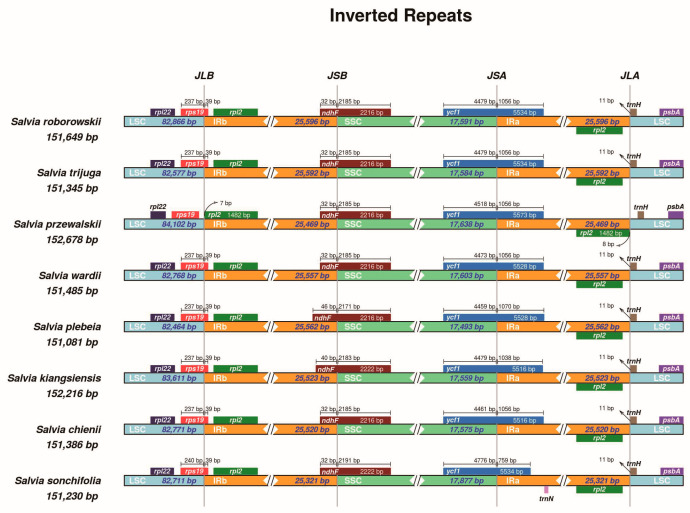
The comparison of the junction positions of LSC, IR, and SSC regions in the chloroplast genomes of the eight *Salvia* species. Genes are annotated in assorted colors and labeled with their distances from the boundaries and the lengths of these distances. JLB, JSB, JSA, and JLA denote the boundaries of the four regions (LSC/IRb, IRb/SSC, SSC/IRa, and IRa/LSC, respectively).

The expansion and contraction of inverted repeat (IR) regions are common evolutionary events in chloroplast genomes that contribute to variations in genome size and gene content. To investigate these structural changes, we compared the boundaries of the LSC, SSC, and IR regions across the eight *Salvia* species. The IR regions in all species were relatively conserved in length, ranging from 25,321 bp to 25,596 bp (Figure 5). Minor variations were observed in the positioning of several genes at the junctions. The *rps19* gene spanned the LSC/IRb (JLB) boundary in all species except *S. przewalskii*, where it was entirely located in the LSC region. In most species, *rps19* had approximately 237–240 bp in the LSC and extended 39 bp into the IRb, forming a partial duplication (*ψrps19*) at the IRa/LSC (JLA) junction. At the SSC/IRa (JSA) boundary, the *ycf1* gene crossed into the IRa, resulting in a truncated pseudogene (*ψycf1*) in the IRb region. The length of *ψycf1* varied slightly among species, ranging from 1038 bp in *S. kiangsiensis*, 10,56 bp in *S. roborowskii*, *S. przewalskii*, *S. trijuga*, *S. wardii*, and *S. chienii*, to 1070 bp in *S. plebeia*, and was as short as 759 bp in *S. sonchifolia*. Similarly, the *ndhF* gene overlapped the IRb/SSC (JSB) boundary, with 32–46 bp extending into the IRb region and the remainder (2171–2191 bp) located in the SSC. The *trnH-GUG* gene, located near the IRa/LSC (JLA) junction, was positioned 11 bp away from the IR boundary in all species except *S. przewalskii*, indicating a subtle but unique boundary shift in this species.

### 3.5. Comparative Chloroplast Genomic Analysis

**Figure 6:**
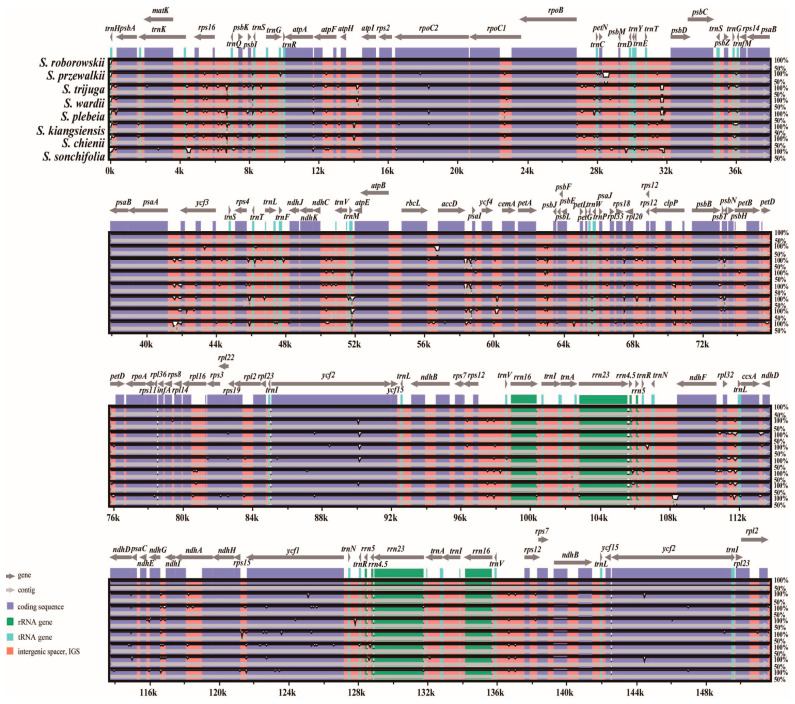
Alignment visualization of the eight *Salvia* cp genomes using *S. roborowskii* annotation as a reference. The horizontal axis indicates the coordinates within the chloroplast genome, and the vertical scale indicates the percentage of identity, ranging from 50% to 100%. Arrows indicate the annotated genes and their transcriptional direction. Genome regions are color-coded as protein coding (purple bars), rRNA coding (green bars), tRNA coding (sky-blue bars), or intergenic regions (IGR, red bars).

**Figure 7:**
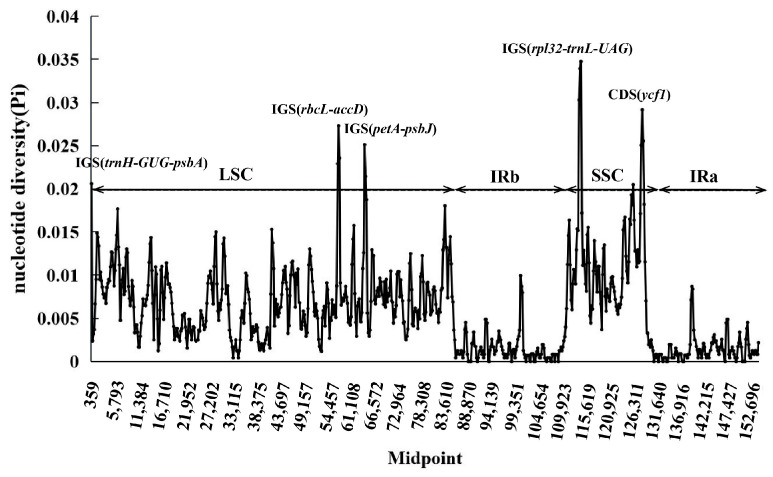
Sliding window analysis of the whole chloroplast genomes of the eight *Salvia* species (window length: 600 bp, step size: 200 bp). *X*-axis: position of the midpoint of a window; *Y*-axis: nucleotide diversity (Pi) in each window.

To assess sequence divergence and structural conservation among the eight chloroplast genomes, a comparative analysis was performed using mVISTA with *S. roborowskii* as the reference (Figure 6). The results revealed a high degree of sequence similarity across the genomes. As expected, the inverted repeat (IR) regions were more conserved than the large single-copy (LSC) and small single-copy (SSC) regions. Additionally, non-coding regions displayed higher sequence variability than coding regions. Several highly variable regions were identified, primarily in intergenic regions (IGR), including *trnH-psbA*, *rps16-trnQ*, *atpI-atpII*, *trnT-psbD*, *psaA-ycf3*, *accD-psaI*, *petA-psbJ*, *rpl32-trnL*) as well as in the *trnM* gene region. To quantify sequence divergence, nucleotide diversity (Pi) values were calculated using a sliding window analysis of DNA polymorphism (Figure 7). The Pi values ranged from 0 to 0.03476, with an overall average of 0.00589. Specifically, nucleotide diversity was highest in the SSC region (0.00369–0.03476; mean = 0.01219), followed by the LSC region (0.00042–0.02732; mean = 0.00727), and lowest in the IR regions (0–0.00994; mean = 0.00149). Five hypervariable loci with Pi values greater than 0.025 were identified: *trnH-psbA* (0.0256), *rbcL-accD* (0.02732), *petA-psbJ* (0.02512), *rpl32-trnL* (0.03476), and *ycf1* (0.02917). Three of these loci were located in the LSC region and two in the SSC, while no highly variable loci were detected in the IR regions, reflecting their structural stability.

### 3.6. Phylogenetic Analyses

**Figure 8:**
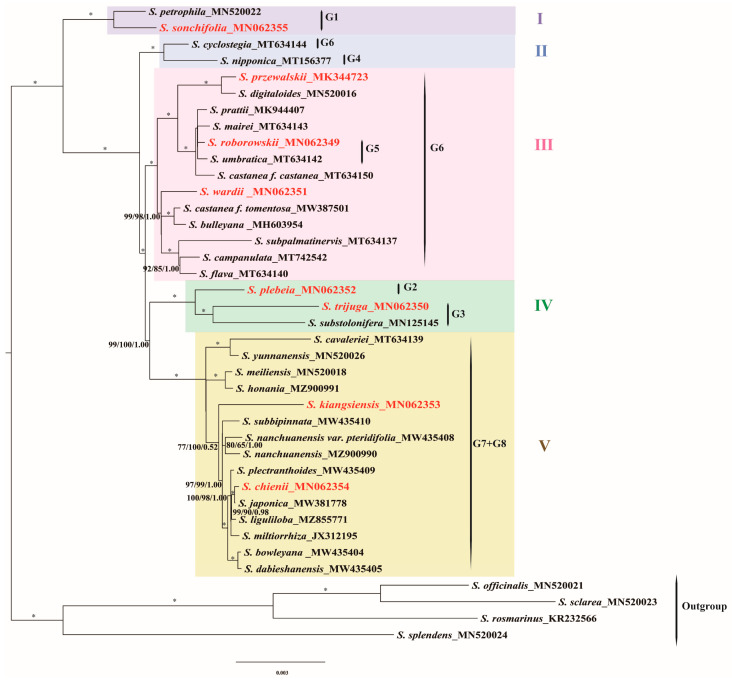
Phylogenetic relationships of the 39 *Salvia* species based on the whole cp genome inferred from maximum likelihood (ML) analyses. The bootstrap support values of ML and MP, and PP values from the BI analysis are listed above the clades, respectively. An asterisk (*) denotes nodes with full support (100% bootstrap/1.00 PP) across all three methods. G1–G8 represent subclades within subgenus *Glutinaria*, as defined by previous taxonomic classifications. Clades I–V represent subclades within subgenus *Glutinaria*, as defined by this study.

The phylogenetic relationships among East Asian *Salvia* species were reconstructed based on complete chloroplast genome sequences using three inference methods: Maximum Parsimony (MP), Maximum Likelihood (ML), and Bayesian Inference (BI). All three methods produced highly congruent topologies with strong statistical support at most nodes. The analyses confirmed the monophyly of East Asian *Salvia* (MLBS = 100%; PP = 1.00; MPBS = 100%) and resolved them into five strongly supported clades (Clades I–V), reflecting distinct evolutionary lineages within the genus (Figure 8). Clade I comprised *S. petrophila* and *S. sonchifolia*, representing a basal lineage sister to the remaining East Asian *Salvia*. These species are limestone endemics sharing unique staminal structures, and *S. sonchifolia* notably retains the ancestral stamen type (Type A) within this group. Clade II contained *S. cyclostegia* (previously identified as the G6 subclade) and *S. nipponica* (previously identified as the G4 clade), grouped together with strong support (MLBS = 100%; PP = 1.00; MPBS = 100%). Both species belonged to the Subg. *Salvia*, sect. *Eurysphace*, and subsect. *Perennes* C. Y. Wu characterized by perennial habits, solitary stems, basal leaves, and corollas typically ranging from 1.5 to 5 cm in length. Clade III encompassed the majority of G6 subclade species (*S. przewalskii*, *S. wardii,* and so on), with two G5 subclade species (*S. roborowskii* and *S. umbratica*) nested within it, reflecting genetic divergence within the clade. Both *S. roborowskii* and *S. umbratica* were classified under the Subg. *Salvia*, sect. *Eurysphace*, and subsect. *Annuae*. They shared characteristics as annual or biennial plants, with stems that were typically much-branched. The leaves were almost entirely cauline and sagittate in shape. The lower portion of the corolla tube was cylindrical and extended beyond the calyx, bending upwards at nearly a right angle. Clade IV grouped *S. plebeia* (previously identified as the G2 subclade) with *S. substolonifera* and *S. trijuga* (previously identified as the G3 subclade), reflecting overlapping geographic distributions and potential evolutionary relationships. Their clustering supports hypotheses of interspecific hybridization and complex divergence in this region. Clade V, the largest and most diverse, included all species from the G7 and G8 subclades (*S. kiangsiensis*, *S. chinenis*, *S. plectranthoides,* and so on). Neither G7 nor G8 formed monophyletic groups independently, but together they represented a distinct clade with high statistical support.

## 4. Discussion

### 4.1. Size and Structure of Chloroplast Genomes

The chloroplast genomes of the eight *Salvia* species examined displayed a typical quadripartite structure, consisting of a large single-copy (LSC) region, a small single-copy (SSC) region, and two inverted repeat (IR) regions. These structural features are consistent with the typical configuration seen in angiosperms and closely align with the average size of land plant chloroplast genomes (151 kb) [1,32]. The GC content ranged from 37.9% to 38.1%, slightly exceeding the average GC content (36.3%) reported in land plant chloroplast genomes [33]. This elevated GC content may enhance genome stability, as GC pairs form three hydrogen bonds (versus two in AT pairs), conferring greater resistance to thermal denaturation and environmental stress [34]. Notably, GC content varied significantly across regions, with the IR region displaying the highest values, likely due to the presence of rRNA genes with reduced AT nucleotide frequency [35,36].

The gene composition, arrangement, and quantity identified within the examined *Salvia* species exhibited a high degree of similarity to the chloroplast genome patterns commonly observed in most angiosperms, as reported in previous studies [37,38]. All eight *Salvia* species examined contained 114 unique chloroplast genes, with no losses in gene content or intron sequences, in agreement with findings in previous studies on other *Salvia* species [16,39,40]. Notably, certain genes, such as *ycf15*, have been reported as absent in *Salvia* species like *S. hispanica*, *S. tiliifolia*, and *S. chanryoenica*, while *rpl32* was found to be absent in *S. splendens* [41]. However, these gene absences were not observed in the eight species of East Asian *Salvia* examined, suggesting potential regional variability in chloroplast gene content. Further investigation is needed to explore the functional and evolutionary roles of the *ycf15* and *rpl32* genes in *Salvia*.

### 4.2. Codon Usage Bias

Codon usage analysis of eight *Salvia* chloroplast genomes revealed a pronounced bias toward A/T-ending codons, in line with the generally AT-rich composition of angiosperm chloroplast genomes [42]. This codon preference was consistent across all species examined, reflecting both evolutionary constraints and potential adaptive roles in chloroplast genome evolution. Notably, a high frequency of A/T-ending codons at the third codon position was observed, particularly for amino acids such as leucine (e.g., UUA and UUG), suggesting a selective advantage for translational efficiency and genome stability, likely shaped by the combined effects of mutation pressure and natural selection. Leucine emerged as the most abundant amino acid in all eight *Salvia* species, while cysteine was the least abundant. Such trends are consistent with findings in other plant families, where codon usage bias not only reflects nucleotide composition but also correlates with protein translation demands. For instance, studies on *Hevea* Aublet and wax gourd chloroplast genomes similarly report an overrepresentation of A/T-ending codons, shaped by a balance of natural selection and mutation pressure to optimize translational accuracy and efficiency [43,44]. Minor interspecies variations, such as the slightly lower leucine codon frequency in *S. sonchifolia*, may indicate subtle adaptations to species-specific environmental or functional demands. Functionally, codon usage bias plays a crucial role in regulating the efficiency and accuracy of protein biosynthesis in chloroplasts. A/T-ending codons are generally associated with higher translational efficiency due to their compatibility with abundant chloroplast tRNA pools, which is especially important under environmental fluctuations that demand rapid protein turnover to maintain photosynthetic performance. Moreover, a bias toward A/T-ending codons can contribute to chloroplast genome stability by minimizing the formation of stable mRNA secondary structures, thus facilitating efficient transcription and translation processes. Comparative analyses in other plant lineages also suggest that codon usage differences can provide insights into evolutionary divergence and adaptation processes [45,46].

### 4.3. LSRs and SSRs Analyses

Long sequence repeats (LSRs) are vital for understanding genome organization, evolutionary dynamics, and structural variations in chloroplast genomes. The identification of 281 LSRs in the eight *Salvia* species, comprising forward, palindromic, tandem, and reverse repeats, highlights their functional diversity. The predominance of forward and tandem repeats is consistent with findings in other *Salvia* chloroplast genomes [47,48], where these repeats contribute significantly to genome rearrangement and mutation hotspots. Most LSRs (85.4%) were shorter than 60 bp, reflecting a preference for smaller repeats that promote recombination while minimizing the risk of deleterious genomic rearrangements. However, the presence of longer repeats (>100 bp, 1.8%) may contribute to structural variations such as inversions, duplications, and deletions, which can increase genome plasticity and provide a substrate for evolutionary innovation under environmental stress. The high concentration of repeats in coding sequences (45.4%) suggests their potential involvement in regulating gene expression, maintaining genome stability, and mediating structural variations within functional genes. Their localization in intergenic (35.4%) and intron regions (8.2%) further underscores their influence on non-coding DNA evolution and possible roles in regulating transcription and RNA splicing efficiency. Conserved repeats in regions such as *rps12-trnV-GAC*, the *ndhA* intron, and *ycf2* likely play a stabilizing role in maintaining genome integrity and facilitating evolutionary conservation across species. Similar conserved repeat elements in *Solanum* and *Mammillaria* chloroplast genomes have been associated with genome stability and phylogenetic signal, acting as indicators of evolutionary processes and divergence history [49,50].

Simple sequence repeats (SSRs), or microsatellites, are critical molecular markers for population genetics, providing insights into genetic diversity, phylogenetic relationships, and genome evolution. In this study, 345 SSRs were identified across the chloroplast genomes of Salvia species, with the counts ranging from 37 in *S. plebeia* to 49 in *S. chienii*. Mononucleotide repeats, predominantly polyadenine (polyA) and polythymine (polyT), constituted the majority (>68.7%), reflecting the AT-rich nature of chloroplast genomes, a trend widely observed in angiosperms [51,52]. Hexanucleotide repeats were rare and detected only in *S. chienii*, while trinucleotide repeats were absent in all species, highlighting species-specific SSR patterns. The concentration of most SSRs in intergenic regions (74.0%) aligns with their role in non-coding DNA evolution, potentially affecting regulatory sequences and contributing to genome size variation. SSR loci were predominantly localized in the large single-copy (LSC) region, consistent with observations in Actinidiaceae and Hamamelidaceae, where SSRs serve as evolutionary hotspots and valuable molecular markers for species delimitation, phylogeography, and chloroplast genome structural evolution [53,54].

### 4.4. Inverted Repeat Expansion and Contraction

The expansion and contraction of inverted repeat (IR) boundaries play a pivotal role in the evolution and structural diversity of chloroplast genomes, often resulting in genome rearrangements and gene duplications, or pseudogenization. In *Salvia*, the boundaries of the LSC, SSC, and IR regions exhibit minor expansions and contractions, with conserved features such as the pseudogenization of *rps19* and *ycf1*. These findings align with studies in *Ligusticum* L. and *Primulina* Hance, which reported similar dynamics at IR boundaries, indicating the conservation of such features across angiosperms [55,56]. The overlap between *ψycf1* and *ndhF* and the stable positioning of *trnH-GUG* suggest functional constraints that preserve chloroplast genome integrity. Comparative analyses reveal that variations in IR boundaries, such as those observed in *Ficus* and Paeoniaceae, are often linked to adaptation to environmental or evolutionary pressures, underscoring their phylogenetic significance [57,58]. The conserved yet flexible nature of IR boundaries in *Salvia* highlights their dual role in maintaining genomic stability while accommodating evolutionary diversification, offering robust markers for phylogenetic studies.

### 4.5. Sequence Divergence and Hypervariable Regions

The comparative analysis of chloroplast genomes in eight *Salvia* species underscores the conserved nature of these genomes while also revealing regions of significant polymorphism that offer insights into evolutionary and phylogenetic dynamics. Consistent with previous studies, our findings confirm that inverted repeat (IR) regions exhibit greater conservation compared to large single-copy (LSC) and small single-copy (SSC) regions, likely due to their structural stability and lower mutation rates [59,60]. The higher variability observed in non-coding regions, particularly in intergenic regions such as *trnH -psbA*, *rbcL-accD*, *petA-psbJ,* and *rpl32-trnL*, aligns with studies on other angiosperms that highlight these regions as hotspots for sequence divergence and potential molecular marker development [61,62]. Our sliding window analysis revealed average nucleotide diversity (Pi) values comparable to those reported in related genera, further substantiating the utility of Pi analysis in pinpointing mutation hotspots for taxonomic resolution [63].

Of particular interest are the loci *ycf1* and the identified intergenic regions, which exhibited Pi values exceeding 0.025, making them prime candidates for barcoding and phylogenetic studies. These loci have been similarly identified in other plant lineages, where they have proven effective in species-level discrimination [64]. Functionally, hypervariable regions may contribute to chloroplast genome adaptability by generating regulatory and coding sequence variation that facilitates responses to environmental pressures. Notably, the absence of significant polymorphism in IR regions corroborates findings in other dicot families [65], where single-copy regions typically harbor the majority of sequence variation. The identification of conserved and highly divergent loci provides a dual advantage: ensuring the stability of broader phylogenetic frameworks while enabling precise species differentiation. Thus, the five hotspots identified here not only advance our understanding of *Salvia* genomic evolution but also hold practical utility in taxonomic classification and evolutionary studies.

### 4.6. Phylogenetic Insights into East Asian Salvia

East Asian *Salvia* species have formally been classified under the subgenus *Glutinaria*, encompassing eight sections (G1–G8), based on molecular analysis (utilizing two nuclear ribosomal spacers and four plastid markers) and morphological investigations [13]. Our results confirm that subgenus *Glutinaria* represents a monophyletic group, strongly supported across three analytical methods. This finding aligns with a series of subsequent studies employing chloroplast genome data [15,16,32]. However, the phylogenetic relationships among the eight sections within subgenus *Glutinaria* remained unresolved. For instance, G1 was identified as the earliest-diverging lineage, followed by the independent divergence of G2. However, the relationships among G3, G4, G5, and G6 remained unclear, while G7 and G8 were identified as sister groups. Later studies identified divergent evolutionary patterns but suffered from limited sampling, representing only a subset of sections (e.g., G1, G4, G6, and G7 in 2020 [15]; G1, G6, G7, and G8 in 2021 [16]; G1, G4, and G6 in 2022 [66]). In this study, we sampled 39 species covering all eight sections of East Asian *Salvia*. The monophyly of G5, G6, and G7 was not supported. Specifically, two species within G5 (*S. roborowskii* and *S. umbratica*) did not cluster together but were embedded within the G6 clade. Similarly, *S. cyclostegia* (G6) and *S. nipponica* (G4) grouped together. Furthermore, three species from G8 were nested within the G7 clade. Monophyly was supported for G1, G3, and G8 but may reflect limited sampling (1–3 species per group).

The basal position of Sect. *Sonchifoliae* (Clade I) within subgenus *Glutinaria* was confirmed, consistent with previous studies [13,32]. This group shares similar leaf morphology and habitats. Notably, stamen type A is found exclusively in *Salvia sonchifolia*. The G2 clade (*S. plebeia*), which was identified as a distinct lineage in Hu et al., 2018, formed a sister group with G3 here (Clade IV), hinting at potential hybridization between these geographically overlapping species, causing discordance between plastid and nuclear phylogenies [13]. G4, G5, and G6 formed Clade II and Clade III; however, their phylogenetic relationships remain unresolved, suggesting that these groups have undergone a relatively complex evolutionary history. The close phylogenetic relationship between G7 and G8 was supported, forming Clade V, encompassing most species traditionally classified under the subgenera *Allagospadonopsis* and *Sclarea* [12]. The observed paraphyly (G7 and G8; G5, and G6) and unresolved relationships may reflect a shared evolutionary history possibly shaped by rapid divergence or recent secondary contact and hybridization. Incomplete lineage sorting remains a plausible explanation as well, especially considering the frequent morphological convergence and overlapping habitats among East Asian *Salvia* species. In summary, our findings suggest that the current sectional classification within East Asian *Salvia* does not fully reflect phylogenetic relationships, and targeted taxonomic revisions are necessary, particularly for G5, G6, G7, and G8. Future studies incorporating nuclear genomic data and morphological reassessment are essential to refine the classification of these groups.

## 5. Conclusions

This study presents a comprehensive comparative analysis of the complete chloroplast genomes of eight East Asian *Salvia* species, providing critical insights into their genetic structure, sequence diversity, and phylogenetic relationships. The chloroplast genomes exhibited a conserved quadripartite structure with genome sizes ranging from 151,081 to 152,678 bp and GC contents from 37.9% to 38.1%. A total of 114 unique genes were consistently identified across all species, reflecting the genomic stability within the genus. Codon usage analysis revealed a pronounced bias toward A/U-ending codons, with leucine as the most frequently encoded amino acid, in line with patterns observed in other angiosperm plastomes. The identification of abundant LSRs and SSRs, predominantly located in non-coding regions, provides a rich resource for developing molecular markers and studying population genetics. Comparative analyses revealed five highly variable regions (*trnH-psbA*, *rbcL-accD*, *petA-psbJ*, *rpl32-trnL*, and *ycf1*), which hold promise as candidate barcodes and molecular markers for phylogenetic, taxonomic, and conservation studies. The phylogenetic analyses supported the monophyly of East Asian *Salvia*, resolving five well-supported clades and confirming the basal position of Sect. *Sonchifoliae*. However, the paraphyly of G5 and G6 and the close relationship between G7 and G8 challenge existing morphological classifications and suggest a complex evolutionary history shaped by hybridization and incomplete lineage sorting. These findings enhance our understanding of chloroplast genome evolution and species relationships within *Salvia*, providing a robust framework for future taxonomic, phylogenetic, and ecological studies. Further research incorporating expanded sampling, nuclear genomic data, and transcriptomic analyses will be essential to fully resolve the evolutionary history of this diverse and economically important genus.

## Figures and Tables

**Table 1:** Information for sampled sequences.

Species	Sample Locality	Voucher	Genbank Accession
*Salvia bowleyana*	Fujian	131	MW435404
*Salvia bulleyana*	na	na	MH603954
*Salvia campanulata*	na	na	MT742542
*Salvia castanea f. castanea*	na	11CS3534	MT634150
*Salvia castanea f.tomentosa*	na	na	MW387501
*Salvia cavaleriei*	na	10CS1700	MT634139
***Salvia chienii***	**Anhui**	**Hu0071**	**MN062354**
*Salvia cyclostegia*	na	HP8813	MT634144
*Salvia dabieshanensis*	Anhui	165	MW435405
*Salvia digitaloides*	Yunnan	1292	MN520016
*Salvia flava*	na	11CS3465	MT634140
*Salvia honania*	na	na	MZ900991
*Salvia japonica*	na	na	MW381778
***Salvia kiangsiensis***	**Jiangxi**	**Hu0062**	**MN062353**
*Salvia liguliloba*	na	na	MZ855771
*Salvia mairei*	na	HP8366	MT634143
*Salvia meiliensis*	Anhui	GX Hu 0089	MN520018
*Salvia miltiorrhiza*	na	na	JX312195
*Salvia nanchuanensis*	na	na	MZ900990
*Salvia nanchuanensis var. pteridifolia*	Guangxi	615	MW435408
*Salvia nipponica*	na	na	MT156377
*Salvia petrophila*	Guizhou	GX Hu 0292	MN520022
***Salvia plebeia***	**Guangxi**	**Hu0024**	**MN062352**
*Salvia plectranthoides*	Yunnan	6	MW435409
*Salvia prattii*	na	na	MK944407
***Salvia przewalskii***	**Yunnan**	**HGW-00807**	**MK344723**
***Salvia roborowskii***	**Gansu**	**FW11193**	**MN062349**
***Salvia sonchifolia***	**Yunnan**	**269**	**MN062355**
*Salvia subbipinnata*	Zhejiang	YJX-04	MW435410
*Salvia subpalmatinervis*	na	YangQE1866	MT634137
*Salvia substolonifera*	na	na	MN125145
***Salvia trijuga***	**Yunnan**	**D576**	**MN062350**
*Salvia umbratica*	na	10CS2479	MT634142
***Salvia wardii***	**Tibet**	**3270**	**MN062351**
*Salvia yunnanensis*	Yunnan	GX Hu QT001	MN520026
*Salvia officinalis*	na	na	MN520021
*Salvia sclarea*	na	na	MN520023
*Salvia splendens*	na	na	MN520024
*Salvia rosmarinus*	na	na	KR232566

Notes: na = not available.

**Table 2:** Basic characteristics of the chloroplast genomes of eight *Salvia* species.

Characteristics	*Salvia* *roborowskii*	*Salvia* *przewalskii*	*Salvia* *trijuga*	*Salvia* *wardii*	*Salvia* *plebeia*	*Salvia* *kiangsiensis*	*Salvia* *chienii*	*Salvia* *sonchifolia*
Genome size (bp)	151,649	152,678	151,345	151,485	151,081	152,216	151,386	151,230
LSC size (bp)	82,866	83,912	82,577	82,768	82,464	83,611	82,771	82,711
IR size (bp)	25,596	25,564	25,592	25,557	25,562	25,523	25,520	25,321
SSC size (bp)	17,591	17,638	17,584	17,603	17,493	17,559	17,575	17,877
Total number of genes	132	132	132	132	132	132	132	132
Protein encoding	80	80	80	80	80	80	80	80
tRNA genes	30	30	30	30	30	30	30	30
rRNA genes	4	4	4	4	4	4	4	4
duplicated genes	18	18	18	18	18	18	18	18
pseudogenes	3	3	3	3	3	3	3	3
GC content (%)	38.0	38.0	37.9	38.0	38.0	38.1	38.0	38.1
GC content of LSC (%)	36.1	36.2	36.0	36.1	36.1	36.3	36.1	36.2
GC content of IR (%)	43.1	43.1	43.1	43.1	43.1	43.1	43.1	43.2
GC content of SSC (%)	31.9	31.9	31.7	31.9	32.0	32.0	32.0	32.0

**Table 3:** A list of genes identified in the chloroplast genomes of eight *Salvia* species.

Category	Gene Type	Gene
Self-replication	rRNA	*rrn16*(2×), *rrn23*(2×), *rrn4.5*(2×), *rrn5*(2×)
	tRNA	*trnI-CAU*(2×), *trnL-CAA*(2×), *trnV-GAC*(2×), * *trnI-GAU*(2×), * *trnA-UGC*(2×), *trnR-ACG*(2×), *trnN-GUU*(2×), *trnL-UAG*, *trnP-UGG*, *trnW-CCA*, *trnM-CAU*, * *trnV-UAC*, *trnF-GAA*, * *trnL-UAA*, *trnT-UGU*, *trnS-GGA*, *trnfM-CAU*, *trnG-GCC*, *trnS-UGA*, *trnT-GGU*, *trnE-UUC*, *trnY-GUA*, *trnD-GUC*, *trnC-GCA*, *trnR-UCU*, * *trnG-UCC*, *trnS-GCU*, *trnQ-UUG*, * *trnK-UUU*, *trnH-GUG*
	Small subunit of ribosome	*rps2*, *rps3*, *rps4*, *rps7*(2×), *rps8*, *rps11*, ** *rps12*(2×), *rps14*, *rps15*, * *rps16*, *rps18*, *rps19*
	Large subunit of ribosome	* *rpl2*(2×), *rpl14*, * *rpl16*, *rpl20*, *rpl22*, *rpl23*(2×), *rpl32*, *rpl33*, *rpl36*
	DNA-dependent RNA polymerase	*rpoA*, *rpoB*, * *rpoC1*, *rpoC2*
Genes for photosynthesis	NADH dehydrogenase	* *ndhA*, * *ndhB*(2×), *ndhC*, *ndhD*, *ndhE*, *ndhF*, *ndhG*, *ndhH*, *ndhI*, *ndhJ*, *ndhK*
	Photosystem I	*psaA*, *psaB*, *psaC*, *psaI*, *psaJ*
	Photosystem II	*psbA*, *psbB*, *psbC*, *psbD*, *psbE*, *psbF*, *psbH*, *psbI*, *psbJ*, *psbK*, *psbL*, *psbM*, *psbN*, *psbT*, *psbZ*
	Cytochrome b/f complex	*petA*, * *petB*, * *petD*, *petG*, *petL*, *petN*
	ATP synthase	*atpA*, *atpB*, *atpE*, * *atpF*, *atpH*, *atpI*
	Large subunit of rubisco	*rbcL*
Other genes	Maturase	*matK*
	Translational initiation factor	*infA*
	Protease	** *clpP*
	Envelope membrane protein	*cemA*
	Acetyl-CoA-carboxylase subunit	*accD*
	c-type cytochrome synthesis	*ccsA*
	Component of TIC complex	*ycf1*
Unknown	Open reading frame (ORF, *ycf*)	*ycf2*(2×), ** *ycf3*, *ycf4*, *ycf15*(2×)

Notes: 2× Genes located in IR region, * Genes with a single intron, ** Genes with two introns.

**Table 4:** The shared repeats of eight *Salvia* species.

No.	Size (bp)	Units	Type	Location Region
1	41	TACAGAACCGTACATGAGATTTTCACCTCATACGGCTCCTC	F	IGR (*rps12*, *trnV-GAC*), *ndhA* (intron)
2	30	A(G)CGGAAAGAGAGGGATTCGAACCCTCGGTA	P	*trnS-GCU* (tRNA), *trnS-GGA* (tRNA)
3	30	CATTGTTCAAA(C)TCTTTGACAACAC(T)GAAAAA	F	IGR (*rrn4.5*, *rrn5*)
4	30	AC(A)GATGCGGGTTCGATTCCCGCTAC(T)CCGCT(C)	F	*trnG-UCC* (tRNA), *trnG-GCC* (tRNA)
5	30	TTTCTTTTTGTCC(G)AAG(C)TCACTTCT(C)TTTTTT	F	*ycf2* (CDS)
6	55	TTTGTCTAAGCCACTTCGTTTCTTTTTGTCCAAGTCACTTCTTTTTTTGTCCAAG	T	*ycf2* (CDS)
7	68	TTTTTGTCCAAGTCACTTCTTTTTTTGTCCAAGTTGCTTTTCTTTTTGTCGAACTCACTTCCTTTTTT	T	*ycf2* (CDS)

## Data Availability

The assembled chloroplast genomes of eight *Salvia* species were deposited in GenBank with the accession numbers of MN062354, MN062353, MN062352, MK344723, MN062349, MN062355, MN062350, MN062351.

## Supplementary Materials

The following supporting information can be downloaded at https://www.mdpi.com/article/10.3390/cimb47070493/s1.

## Abbreviations

The following abbreviations are used in this manuscript:

LSC	Large single-copy
SSC	Small single-copy
IR	Inverted repeat sequence
GC	Guanine/cytosine content
PCG	Protein-coding gene
SSRs	Simple sequence repeats
LSRs	Long sequence repeats
BI	Bayesian inference
ML	Maximum likelihood
MP	Maximum Parsimony
CP	Chloroplast
IGR	Intergenic regions
PP	Posterior probability
